# To get sick or not to get sick—*Trichomonas* infections in two *Accipiter* species from Germany

**DOI:** 10.1007/s00436-021-07299-1

**Published:** 2021-09-04

**Authors:** Manuela Merling de Chapa, Susanne Auls, Norbert Kenntner, Oliver Krone

**Affiliations:** 1 Leibniz Institute for Zoo and Wildlife Research, Department of Wildlife Diseases, Alfred-Kowalke-Straße 17, 10315 Berlin, Germany; 2 Independent Researcher, Berlin, Germany

**Keywords:** *Trichomonas gallinae*, Parasitic infection, Birds of prey, Host-parasite coevolution, Naïve host, Trichomonosis

## Abstract

Trichomonosis caused by the flagellate *Trichomonas gallinae* is one of the most important avian diseases worldwide. The parasite is localised in the oesophageal area of its host and mainly infects pigeon and dove species. During the last decade, a host expansion to passerine birds occurred, making the disease a potential threat for passerine predators as naïve host species. Here, we investigated the effect of the parasite on two *Accipiter* species in Germany which show a comparable lifestyle but differ in prey choice, the Northern goshawk (*Accipiter gentilis*) mainly hunting pigeons and the Eurasian sparrowhawk (*Accipiter nisus*) mainly feeding on passerines. We genetically identified the parasite strains using the Fe-Hydrogenase gene as marker locus and compared the incidence of parasite presence and clinical signs of trichomonosis between nestlings of the two *Accipiter* species. In total, we identified 14 strains, with nine strains unknown so far. There was a higher strain diversity and prevalence of *Trichomonas* spp. in goshawks than sparrowhawks (42.4% vs. 21.2%) whereas sparrowhawks when being infected more often displayed clinical signs of trichomonosis than goshawks (37.1% vs. 6.1%). Even though sparrowhawks were mainly infected with the finch epidemic strain and genetic data indicated some variation between isolates, no correlation with virulence could be detected. All in all, goshawks seem to be better adapted to *Trichomonas i*nfections, whereas to sparrowhawks, this is a novel disease with more severe manifestations, from individual morbidity to a higher risk of population decline caused by trichomonosis.

## Introduction

Avian trichomonosis caused by the flagellate *Trichomonas* spp., mainly by *Trichomonas gallinae* (Rivolta 1878), is considered a major disease of numerous avian species, especially Columbiformes and Accipitriformes, and has been reported from several continents (Stabler 1954). The rock pigeon (*Columba livia*, Gmelin 1789) is the primary host of *T. gallinae* and considered responsible for the worldwide distribution of this protozoan (Stabler 1954; Harmon et al. 1987). The parasite is located in the upper digestive and rarely in the respiratory tract of the host and transmitted via a direct life cycle (Forrester and Foster 2008). In pigeons, the parasite is transmitted vertically due to crop milk feeding (Stabler 1954; Kocan and Herman 1971; Forrester and Foster 2008) and horizontally due to billing (Kocan and Herman 1971). Interspecies transmission can occur when predators feed on infected prey or at shared feeding and drinking places because of contaminated food or water (Kocan 1969; Forrester and Foster 2008; Lawson et al. 2012; McBurney et al. 2017). The effects of the infection vary from subclinical to substantial clinical symptoms that lead to tissue necrosis, caseation, invasion of inflammatory cells and death of the host (Kocan and Herman 1971; Forrester and Foster 2008). Avian trichomonosis has been recognised as an emerging infectious disease of wild finches in the UK (Robinson et al. 2010) which further spreads as a consequence of bird migration (Lawson et al. 2011b). Such disease emergence usually occurs via the introduction of a novel pathogen into a naïve host population, resulting in increased morbidity and mortality (Daszak et al. 2000; Williams et al. 2002). The severity of the disease depends on the susceptibility of the infected birds as well as the virulence of the incriminated strain (Cooper and Petty, 1988; Cole et al. 1999). Different strains of *T. gallinae* vary in their pathogenicity (Stabler 1954; Forrester and Foster 2008). Therefore, it is important to characterise the parasite strains in order to determine potential effects at the population level (Quillfeldt et al. 2018). Genetic characterisation of the parasite gives insights into the ecology and epidemiology of avian trichomonosis and refines the understanding of parasite-host associations and virulence of different strains (Sansano-Maestre et al. 2009; Rogers et al. 2016). Sequences of the Fe-hydrogenase region are often used to identify different *T. gallinae* strains because of its fast evolution, allowing a fine-scale characterisation of strains (Chi et al. 2013; Alrefaei et al. 2021). On the basis of this genetic characterisation, the single clonal strain A1 was detected as the causative agent of this emerging infectious disease and the epidemic passerine mortality (Lawson et al. 2011a).

The disease trichomonosis is described to have an enormous impact on numerous wild bird populations (Boal et al. 1998; Robinson et al. 2010; Lawson et al. 2011b; Amin et al. 2014; Merling de Chapa et al. 2020). However, the overall prevalence of *Trichomonas* spp. in many wild bird populations remains mostly unclear. Prospective studies monitoring the exposure to parasites in wild bird populations are helping to better quantify and understand the parasite’s effect on population dynamics (Hochachka and Dhondt 2000).

In this study, we screened different *Accipiter* populations for the presence of *Trichomonas* spp. in Northern Germany. The proportion of the diet that consists of avian prey and the relative importance of columbid and passerine prey vary amongst raptors (Chi et al. 2013). Therefore some predators are likely to have been exposed to *Trichomonas* for a long time and may have responded to them in terms of a co-evolved host-parasite relationship, whereas to others, this is a novel challenge for which they are little prepared and thus expected to be at a greater risk to infection from trichomonosis (Lawson et al. 2012; Chi et al. 2013). Here, we focused on two raptor species that are believed to be at special risk to get infected with *Trichomonas* spp., the Northern goshawk (*Accipiter gentilis*, Linnaeus 1758) and the Eurasian sparrowhawk (*Accipiter nisus*, Linnaeus 1758). Both species mainly hunt avian prey (Newton 1986; Kenward 2006). The goshawk mainly hunts middle-sized bird species, especially rock pigeons (*C. livia*) and their feral relatives (*Columba livia f. domestica*, Gmelin 1789) and has inhabited urban areas in the last decades (Kenward 2006). The threatening role of trichomonosis seems to be particularly important for birds of prey that nest in or near urban areas (Boal 1997; Boal et al. 1998; Amin et al. 2014). In a recent study, we showed that prevalence of *T. gallinae* in Northern goshawks was higher in the urban environment than in the rural one (Merling de Chapa et al. 2020). A high prevalence with *T. gallinae* was observed in urban breeding goshawks, with a prevalence of 100% in Poland (Wieliczko et al. 2003) and 65% in Germany (Krone et al. 2005) as well. Krone et al. (2005) stated that the combination of a high prevalence of *T. gallinae* in Northern goshawks with a low incidence of clinical symptoms seems to be the result of a co-evolutionary host-parasite relationship.

The Eurasian sparrowhawk has a high probability of encountering the parasite as well (Kunca et al. 2015), since small and medium-sized birds comprise 97% of its diet during the breeding season (Newton 1986) of which 76% consists of passerine birds (Cotgreave 1995). A breeding bird survey in the UK demonstrated a significant decline in the sparrowhawk populations with onset in 2006 (Baillie et al. 2007), which is simultaneous with the emergence of epidemic finch trichomonosis (Robinson et al. 2010; Lawson et al. 2011a). Peters et al. (2009) reported the emergence of finch trichomonosis in Northern Germany in April 2009. The possibility that this disease may have affected the Eurasian sparrowhawk population in this area therefore requires urgent investigation (Chi et al. 2013).

The objective of our study is to analyse how the two *Accipiter* species are influenced by *Trichomonas* spp., especially *T. gallinae*, the most common agent of the disease trichomonosis. Therefore, we monitored parasite prevalence in different goshawk and sparrowhawk populations from Germany, genetically characterised the occurring parasite strains and analysed the impact of the parasite on the host populations to answer the following questions:

1. With which strains are the raptors infected?
2. Do Northern goshawks show a higher prevalence with the parasite *Trichomonas* spp. due to a higher encounter probability because of the high amount of pigeons in their diet compared to Eurasian sparrowhawks?
3. Do Eurasian sparrowhawks have a higher chance than Northern goshawks to show clinical signs of the disease?
4. Can we find a link between the incidence of clinical signs and the identity of parasite strains to assess virulence of strains?

## Material and methods

### Study sites and samples

The study was carried out between 2014 and 2016 at seven study sites in Northern Germany (Merling de Chapa et al. 2020). The Northern goshawks were sampled in urban and rural sites, the Eurasian sparrowhawks were just sampled in rural locations while we were not able to access sparrowhawks at our urban locations. The urban sites were three cities with human populations of more than 1 million each: Berlin, Cologne and Hamburg. The rural sites were located in the area of Barnim, the lower Rhine area near Kleve in an area close to the border between Germany and Netherlands, rural areas around Schleswig as well as areas inside the Teutoburg forest around Bielefeld (Merling de Chapa et al. 2020). Eurasian sparrowhawks were sampled in two rural forests: in the region of Kleve and, in the year 2015, also in the region of Barnim. Some territories were investigated over several study years.

#### Field work

The study was carried out on raptor nestlings which were examined during banding between May and June of each study year. Nestlings were collected from the nest, handled on the ground and later returned to the nest. The body mass of a nestling combined with its wing-length measurement allowed us to distinguish males and females, as well as determine nestling age (Bijlsma 1997). In total, we sampled 545 goshawk nestlings in 197 nests from 134 different breeding territories as well as 165 sparrowhawk nestlings in 43 nests from 35 different breeding territories. Most sparrowhawk samples (*n* = 152) came from the region of Kleve.

All nestlings were tested for the presence of the endoparasite *Trichomonas* spp. by taking a sample from the oro-pharyngeal region using sterile cotton swabs. After sampling, swabs were stored in a special culture medium (InPouch TV, Biomed Diagnostics, White City, USA) and placed in a mobile incubator at 37 °C for an incubation period of 10 days. Starting on the fifth day of incubation, the presence of the flagellate parasite was checked every day using a light microscope at × 100 and × 200 magnification. We considered samples to be negative when no parasite had been observed during the incubation period. When the parasite could be detected, individuals were called infected with *Trichomonas* spp. including asymptomatic cases and symptomatic cases. To distinguish these cases, all nestlings were examined for clinical signs of the disease trichomonosis by checking the oesophageal region of the nestlings for the presence of yellowish plaques during sampling. The calculations for the amount of nestlings showing clinical signs of the disease are related only to the amount of nestling tested positive for the presence of *Trichomonas* spp..

### Genetic characterisation of parasite strains

We excluded one goshawk territory with three nestlings from further analysis, as here the nestlings showed strong clinical signs of an oesophageal disease but no trichomonad could be detected. The clinical signs could have been caused by *Salmonella* spp. which is known to induce similar signs (Lawson et al. 2010) or stomatitis from other causes (Krone et al. 2005). Therefore, in this study, 230 out of 542 goshawks and 35 out of 165 sparrowhawks were positive for *Trichomonas* spp.

The InPouch media containing *Trichomonas* spp. parasites were split into two subsamples, centrifuged at 10,000 × g for 5 min and the supernatant was discarded. The pellets were washed twice with PBS. One pellet was stored at − 80 °C as a retain sample, the other one was used for further DNA extraction. DNA was extracted using the DNeasy Blood & Tissue Kit (Qiagen, Hilden, Germany) according to the manufacturer’s instruction, with the exception that the DNA was eluted in 30 µl of buffer.

To examine the Fe-hydrogenase gene of *Trichomonas* spp., two different primer pairs were used. Since the widely used primers described in Lawson et al. (2011a) did not produce a fragment in many of our samples, we performed the PCR with the primer pairs FehydFOR6 (5′-CTGCTCTGAAGAGGGCATCG-3′)/FehydREV3 (5′-GTCTGTCTCCTTGAGGCCAG-3′) and FehydFOR9 (5′-ACATGAACGTCGCCTACTCCG-3′)/FehydREV5 (5′-TGTYTCCTTGRGGCCAGWCTTTG-3′) located within the fragment described by Lawson et al. (2011a). Based on the reference sequence JF681136 (Lawson et al. 2011a), the fragment FehydFOR6/FehydREV3 is located between position 133 and 851 and the fragment FehydFOR9/FehydREV5 is located between position 91 and 845. The primer pair FehydFOR9/FehydREV5 produced fragments with higher probability. However, when we had already produced a fragment with FehydFOR6/FehydREV3, we did not perform a new amplification with the other primer pair, but instead used this fragment for sequencing.

PCR amplification of the ITS1/5.8S rRNA/ITS2 region was performed with those samples in which we detected a novel strain in the Fe-hydrogenase gene to ensure correct assignment of the novel strains. PCR was performed using TFR1 (5′-TGCTTCAGCTCAGCGGGTCTTCC-3′) and TFR 2 (5′-CGGTAGGTGAACCTGCCGTTGG-3′) primers (Felleisen 1997).

The PCR was conducted using the FastStartTaq DNA Polymerase dNTPack (Roche Diagnostics, Mannheim, Germany). PCR mixtures contained 1 × PCR Reaction Buffer, 1.5 mM MgCl_2_, 0.2 mM dNTP mix, 1 U FastStart Taq DNA Polymerase, 0.24 µM of each primer, and 50 ng of DNA in a final volume of 25 µl. A negative control of water and a positive control of purified *T. gallinae* DNA from a greenfinch (*Chloris chloris*, Linnaeus 1758) were included in each PCR run. Reaction mixtures were subjected to the following PCR cycling protocol: 94 °C for 4 min, 35 cycles of 94 °C for 1 min, 50 °C for 1 min and 72 °C for 1 min, followed by 72 °C for 5 min. The PCR products were purified (ExoSAP; Thermo Fisher Scientific, Waltham, USA), directly sequenced using the BigDye Terminatorv1.1 Cycle Sequencing kit (Thermo Fisher Scientific, Waltham, USA) with the PCR primers and analysed on a 3130xl Genetic Analyzer (Thermo Fisher Scientific, Waltham, USA). Raw sequences were manually inspected and refined using MEGA6 (Tamura et al. 2013). In cases of a double infection, shown by interfering sequences, the affected amplicons were cloned using the TOPO TA Cloning® Kit for Sequencing with One Shot® TOP10 Chemically Competent *E. coli* (Invitrogen, Glasgow, UK) according to the manufacturer’s instructions. Ten clones per PCR product were selected and transferred to 20 μl of water. The clones were used as template in a PCR as published by Andree et al. (2013). The PCR products were purified, and Sanger sequenced as described above. Plausibility of results was verified in certain cases using the retained sample e.g. the presence of different strains in the same nest.

**Fig. 1:**
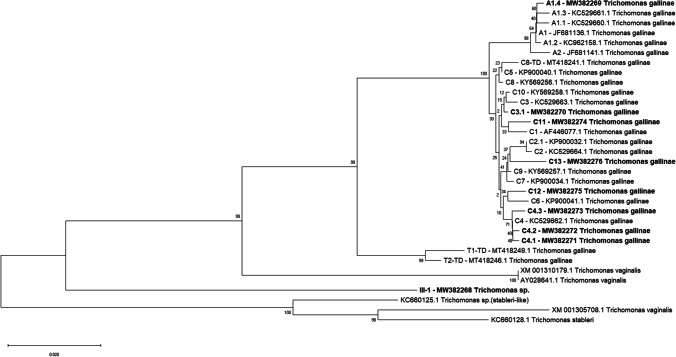
Strains of the Fe-hydrogenase gene region. References to GenBank accession numbers are: AF446077, AY028641.1 (Voncken et al. 2002); JF681136.1, JF681141.1 (Lawson et al. 2011a); KC529660.1-KC529664.1, KC962158.1 (Chi et al. 2013); KC660125.1, KC660128.1 (Girard et al. 2014a); KP900032.1, KP900034.1, KP900040.1, KP900041.1 (Sansano-Maestre et al. 2016); KY569256.1-KY569258.1 (Alrefaei AF, direct submission to GenBank 2017); MT418241.1, MT418246.1, MT418249.1 (Dunn et al., direct submission to GenBank 2020); XM001305708.1, XM001310179.1 (Carlton et al. 2007). Novel strains found in this study are A1.4, C3.1, C4.1, C4.2, C4.3, C11, C12, C13, III-1 and marked in **bold**

Fe-hydrogenase gene sequences from this study were aligned with published *Trichomonas* spp. sequences obtained from the NCBI GenBank database using Geneious (Geneious Prime 2019.2, http://www.geneious.com). A single consensus sequence was used to represent identical sequences in phylogenetic analyses. Novel sequences were submitted to GenBank (MW382268-MW382276). The evolutionary history was inferred using the neighbour-joining method (Saitou and Nei 1987). In Fig. 1, the optimal tree is shown. The tree is drawn to scale, with branch lengths in the same units as those of the evolutionary distances used to infer the phylogenetic tree. The evolutionary distances were computed using the maximum composite likelihood method (Tamura et al. 2004) and are in the units of the number of base substitutions per site. Only the overlapping range of sequences was used for the analysis. There was a total of 711 positions in the final data set. However, the sequences KC5296631.1, MT418241.1, MT418246.1 and MT418249.1 from GenBank were included even though they covered only 590 bp of the analysed region. For this, all ambiguous positions for each pair of sequences were removed (‘pairwise deletion’ option). The analysis included 33 nucleotide sequences. Codon positions 1 + 2 + 3 were considered. Evolutionary analyses were conducted in MEGA X (Kumar et al. 2018).

### Prevalence and clinical signs of the parasitic infection

#### Statistical analyses

We analysed the effect of different covariates on the presence of *Trichomonas* and the presence of clinical signs of the disease by means of generalised linear mixed-effects models (GLMM). All statistical analyses were performed using R version 4.0.3 (R Core Team, 2020). To investigate the presence of *Trichomonas*, we used logistic (binary) GLMM with the presence/absence of *Trichomonas* spp. as response variable. To investigate the effect of age and condition on our response variables, we measured condition via a linear regression analysis using the least squares method of wing length and weight calculating the standard residuals. These standard residuals were used as condition covariate. For the age of nestlings, we categorised individuals into young, middle and old nestlings for goshawks between 11 and 19 days, 20 and 27 days and 28 and 40 days, and for sparrowhawks between 3 and 10 days, 11 and 17 days and 18 and 23 days, respectively. These calculations allowed us to use comparable quantities between species and sexes. In a previous study, we showed that urban goshawks show a higher prevalence of the parasite *T. gallinae* than rural ones (Merling de Chapa et al. 2020). To control for the possible influencing factor that the environment could bear and to address the issue that sparrowhawks were only sampled in the rural environment, we divided goshawks into ‘urban goshawks’ and ‘rural goshawks’. Covariates included in this model were species (categorical, three categories: ‘goshawk urban’, ‘goshawk rural’, ‘sparrowhawk’), year (categorical, three categories: ‘2014’, ‘2015’, ‘2016’), sex (categorical, two categories: ‘female’, ‘male’), condition (continuous, values from -3.709 to 3.356) and age (categorical, three categories: ‘young’, ‘middle’, ‘old’). We considered the territory (168 levels) and the location (7 levels) as random predictors to account for multiple measurements and spatial autocorrelation. In a second step, we summarised the urban and rural goshawks with a link function to the overall variable ‘goshawk’ to investigate the effect that the species (categorical, two categories: ‘linked goshawk’, ‘sparrowhawk’) had on the chance to get infected.

We identified factors influencing the chance to develop clinical signs of trichomonosis with the help of a logistic (binary) GLMM, with the presence/absence of clinical signs as response variable. To simplify the model, we only included the species (categorical, two categories: ‘goshawk’, ‘sparrowhawk’), the year (categorical, three categories: ‘2014’, ‘2015’, ‘2016’), the sex (categorical, two categories: ‘female’, ‘male’) and the strain category (categorical, four categories: ‘A1’, ‘C4’, ‘different’, ‘double’) as predictors. Strains of categories ‘A1’ and ‘C4’ also contained the subtypes of these strains, ‘different’ included all other strains and ‘double’ was categorised as individuals being infected with two strains. We considered the territory (66 levels) as random predictor to account for multiple measurements. We used a Fisher’s exact test, as implemented by the function fisher.test in R, to test the effect of all single strains on the chance to develop clinical signs of the disease for goshawks and sparrowhawks (without controlling for any confounding variables), ignoring cases of double infections. Another Fisher’s exact test was conducted to test for the effect of double infections.

All models were fitted using the package spaMM 3.5.32 (Rousset and Ferdy 2014). We checked that the main assumptions of linear modelling (lack of serial autocorrelation, expected dispersion and distribution of residuals) were fulfilled using DHARMa 0.3.3 (Hartig 2019). We computed the significance of fixed-effect parameters in all models using a likelihood ratio test: we compared the observed LRT statistic (hereafter *χ*^2^) to its distribution under the null hypothesis to compute the *p*-value. The latter distribution and the *p*-value were obtained through 1000 parametric bootstraps using the function *anova*() from spaMM.

## Results

### Genetic characterisation of parasite strains

#### Fe-hydrogenase gene

We obtained 247 Fe-hydrogenase gene sequences of variable length (718–754 bp) from 221 individuals consisting of 189 goshawk and 32 sparrowhawk isolates. Of these birds, 26 (20 goshawks and six sparrowhawks) were infected with two different strains, which we call double infection. No PCR fragment could be amplified from 44 microscopically positive detected samples.

In total, we found 14 different strains in the two *Accipiter* species analysed (Fig. 1). Occurrence of individual strains differed between species as well as between the locations (Table 1). In total, goshawks were infected with 13 strains (11 in urban and 9 in rural environments) and sparrowhawks with five.

**Table 1:** Proportion of Trichomonas strains per single location. *ACGE* goshawk, *ACNI* sparrowhawk

Species	Location (*n* = number of strains)	Strains (in %)													
		A1	A1.2	A1.4	A2	C3.1	C4	C4.1	C4.2	C4.3	C7	C11	C12	C13	III-1
ACGE	Berlin (*n* = 111)	23.4	0.0	0.0	4.5	0.0	61.3	9.9	0.0	0.0	0.0	0.9	0.0	0.0	0.0
	Hamburg (*n* = 27)	48.1	3.7	7.4	3.7	0.0	7.4	0.0	11.1	0.0	0.0	0.0	0.0	14.8	3.7
	Cologne (*n* = 18)	44.4	0.0	0.0	0.0	5.6	33.3	0.0	16.7	0.0	0.0	0.0	0.0	0.0	0.0
	**Urban** (*n* = 156)	30.1	0.6	1.3	3.8	0.6	48.7	7.1	3.8	0.0	0.0	0.6	0.0	2.6	0.6
	Barnim (*n* = 9)	22.2	0.0	0.0	0.0	0.0	77.8	0.0	0.0	0.0	0.0	0.0	0.0	0.0	0.0
	Schleswig (*n* = 6)	83.3	0.0	0.0	0.0	0.0	16.7	0.0	0.0	0.0	0.0	0.0	0.0	0.0	0.0
	Bielefeld (*n* = 10)	50.0	0.0	0.0	20.0	10.0	20.0	0.0	0.0	0.0	0.0	0.0	0.0	0.0	0.0
	Kleve (*n* = 28)	25.0	3.6	0.0	0.0	0.0	53.6	0.0	0.0	3.6	0.0	3.6	7.1	3.6	0.0
	**Rural** (*n* = 53)	35.8	1.9	0.0	3.8	1.9	47.2	0.0	0.0	1.9	0.0	1.9	3.8	1.9	0.0
	**Total** (*n* = 209)	31.6	1.0	1.0	3.8	1.0	48.3	5.3	2.9	0.5	0.0	1.0	1.0	2.4	0.5
ACNI	Barnim (*n* = 4)	100	0	0	0	0	0	0	0	0	0	0	0	0	0
	Kleve (*n* = 34)	64.7	0	0	0	0	14.7	14.7	0	0	2.9	0	0	2.9	0
	**Total** (*n* = 38)	68.4	0	0	0	0	13.2	13.2	0	0	2.6	0	0	2.6	0

The finch epidemic strain A1 (Lawson et al. 2011a) was the most common strain in sparrowhawks with 26 out of 38 isolates (68.4%), and the second most common strain in goshawks with 66 out of 209 individuals (31.6%) being infected with it (Table 1). One nest of urban goshawks showed an isolate that varied from A1 in one single nucleotide polymorphism (SNP) and was therefore termed A1.4.

We identified C4 as the most common strain within goshawks with 101 out of 209 isolates (48.4%), and 5 out of 38 isolates (13.2%) in sparrowhawks (Table 1). Three subtypes of strain C4 were found, which varied from C4 by one SNP, and were therefore termed C4.1, C4.2 and C4.3. We found a subtype of the strain C3 described in Chi et al. (2013). It differed from C3 in two SNP and was termed C3.1. We also identified three novel strains which could be phylogenetically assigned to type C (Fig. 1) and called them C11, C12, C13. One strain (GenBank accession number MW382268 = sample ID 3,113,044) differed phylogenetically much more from the other strains. This strain did not belong to *T. gallinae* and was more closely related to *Trichomonas stableri* (Girard et al. 2014a) (Fig. 1).

#### ITS1/5.8S rRNA/ITS2 region

We obtained 38 nucleotide sequences a 327 bp from the ITS1/5.8S rRNA/ITS2 region from all those samples which we identified as a novel (Fe-hydrogenase) strain to better characterise these strains. Both samples with Fe-hydrogenase strain A1.4 were identical to GenBank sequence EU215369 characterised as ITS strain A (Gerhold et al. 2008). All 35 samples with Fe-hydrogenase strains C3.1, C4.1, C4.2, C4.3, C11, C12 and C13 were identical to GenBank sequence EU215362 characterised as ITS strain C (Gerhold et al. 2008). The *Trichomonas* spp. sample 3,113,044 was identical to GenBank sequence FN433473.1 and was thereby identified as ITS type III (Grabensteiner et al. 2010). Therefore, we called this strain III-1.

Raptor nestlings from the same nest did not always have the same strains. In 24 of 85 goshawk nests and three of 11 sparrowhawk nests, the nestlings had different strains.

### Prevalence and clinical signs of the parasitic infection

Overall, 265 out of 707 individuals (37.5%) were infected with *Trichomonas* spp. (Table 2) including asymptomatic and symptomatic cases.

**Table 2:** Prevalence of *Trichomonas* presence and of clinical signs of trichomonosis at different sampling locations in both *Accipiter* species. *ACGE* goshawk, *ACNI* sparrowhawk

Species	Location	Infected (*N*_total_ = all birds sampled)			Clinical signs (*N*_infected_ = number of infected birds)		
		Yes	*N*_total_	%_infected_	Yes	*N*_infected_	%_clinical signs_
ACGE	Berlin	116	178	65.2	6	116	5.2
	Hamburg	26	45	57.8	3	26	11.5
	Cologne	18	62	29	2	18	11.1
	**Urban**	160	285	56.1	11	160	6.9
	Barnim	16	42	38.1	3	16	18.8
	Schleswig	12	62	19.4	0	12	0
	Bielefeld	11	42	26.2	0	11	0
	Kleve	31	111	27.9	0	31	0
	**Rural**	70	257	27.2	3	70	4.3
	**Total**	230	542	42.4	14	230	6.1
ACNI	Kleve	31	152	20.4	13	31	41.9
	Barnim	4	13	30.8	0	4	0
	**Total**	35	165	21.2	13	35	37.1

The odds of a goshawk nestling to be infected with *Trichomonas* spp. was in total 4.7 times higher than for sparrowhawk nestlings. Specifically, 230 of 542 (42.4%) goshawk nestlings tested positive for the parasite, 35 of 165 (21.2%) sparrowhawk nestlings did so (Table 2). This effect was significant in a GLMM accounting for other variables (GLMM, *n* = 696: *χ*^2^ = 9.08, *p* = 0.048). The odds that an urban goshawk nestling got infected was 9.5 times higher than for a sparrowhawk nestling, whereas the chance for a rural goshawk nestling was only 2.2 times higher.

**Fig. 2:**
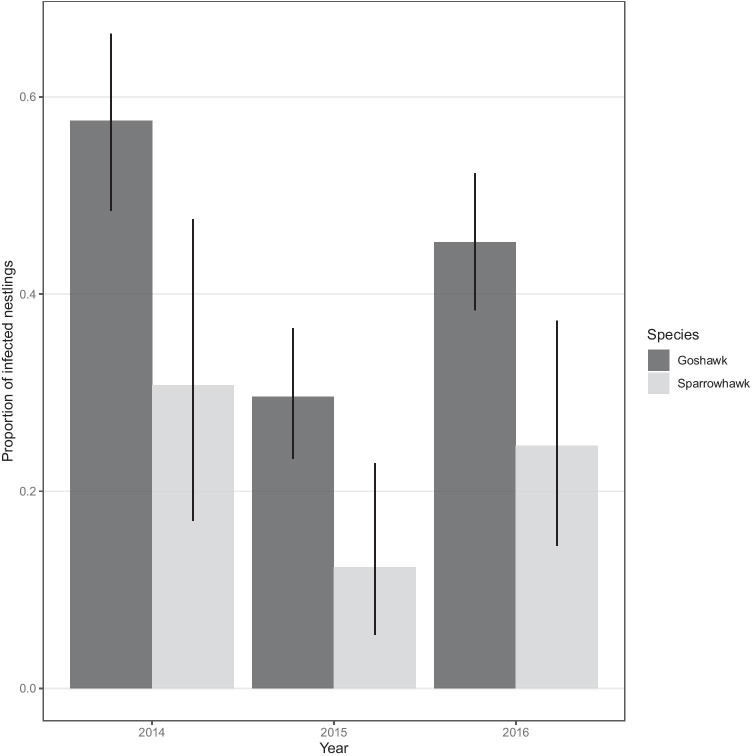
Proportion of infected goshawk (dark grey) and sparrowhawk (light grey) nestlings (± CI_95%_) between the three sampling years

**Fig. 3:**
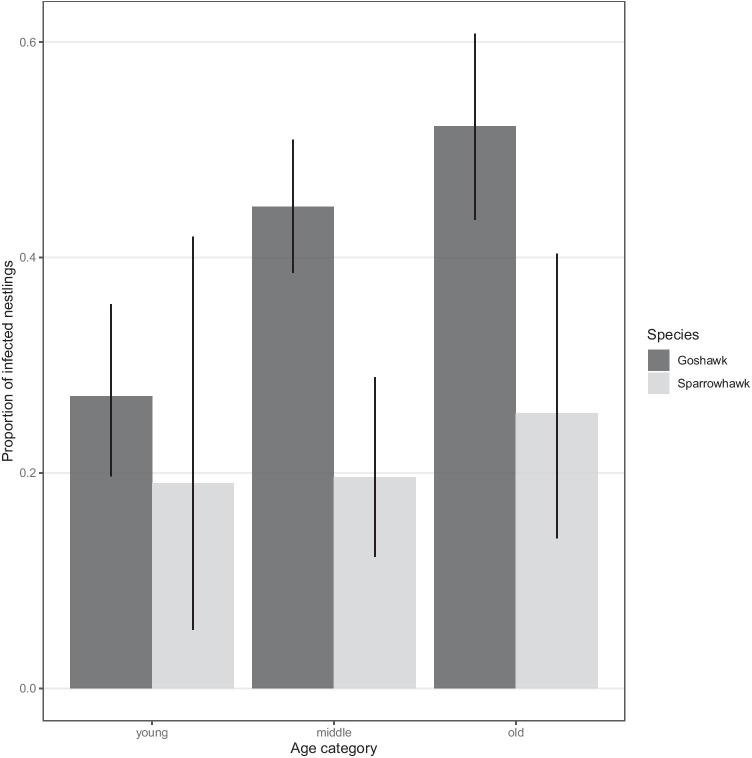
Proportion of infected goshawk (dark grey) and sparrowhawk (light grey) nestlings (± CI_95%_) between the three age categories

Prevalence varied significantly between sampling years (*χ*^2^ = 24.8, *p* < 0.001), being considerably lower in 2015 than in 2014 and 2016 (Fig. 2). Prevalence significantly increased with nestling age as well (*χ*^2^ = 8.24, *p* = 0.026) (Fig. 3). Other independent variables did not significantly predict the prevalence (sex: *χ*^2^ = 0.894, *p* = 0.366; condition: *χ*^2^ = 0.157, *p* = 0.692).

In total, 27 of 265 infected individuals showed clinical signs of trichomonosis (6.4%). Specifically, 14 of 230 (6.1%) infected goshawks and 13 of 35 (37.1%) infected sparrowhawks showed clinical signs of the disease (Table 2). Sparrowhawks were 6.2 times more likely to show clinical signs of trichomonosis than goshawks (GLMM, *n* = 216: *χ*^2^ = 4.91, *p* = 0.048). Nevertheless, this finding should be interpreted with caution because of the low number of individuals showing clinical signs. Other independent variables did not significantly predict clinical outcome (sex: *χ*^2^ = 0.251, *p* = 0.632; year: *χ*^2^ = 3.75, *p* = 0.169).

**Fig. 4:**
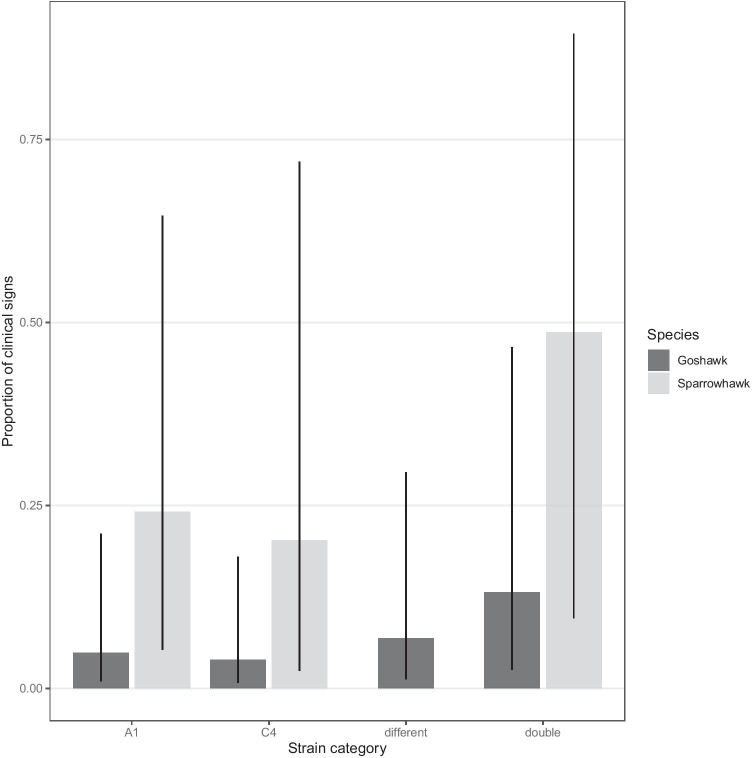
Predicted probability of clinical signs connected to strain categories between infected goshawk (dark grey) and sparrowhawk (light grey) nestlings. Error bars show CI_95%_

The strain category did not seem to affect the chance to develop clinical signs in goshawks or sparrowhawks (Table 3, Fig. 4). A Fisher’s exact test did not find a relationship between strain identity and the chance of clinical signs (goshawks: *p* = 0.069, sparrowhawks: *p* = 0.093). Also, the GLMM testing for the importance of other covariates did not detect any effect of the strain category on the chance to show clinical signs of trichomonosis in goshawks or sparrowhawks (GLMM, *χ*^2^ = 3.34, *p* = 0.386, *n* = 216, Fig. 4). However, a Fisher’s exact test predicted a significant influence of double infections, and *Accipiter* with a double infection were 4.6 times more likely to develop clinical signs (Fisher’s exact test: *p* = 0.003). This effect can be seen in the predictive plot of the GLMM as well (Fig. 4).

**Table 3:** All individuals showing clinical signs of the disease. *ACGE* goshawk, *ACNI* sparrowhawk, *nA* not available (no PCR fragment could be amplified from these microscopical positive samples)

Species	Year	Location	Territory ID	Individuals	Strain
ACGE	2014	Berlin	1	JC68873	C4
		Berlin	1	KT1613	C4
		Berlin	2	E12	A1
	2015	Berlin	3	D61	C4/C11
		Berlin	4	E15	A2
		Barnim	5	EA189435	A1/C4
		Barnim	6	EA189454	nA
		Barnim	6	EA189455	nA
		Hamburg	7	N110927	A1.4
	2016	Berlin	1	F86	C4
		Hamburg	8	N110405	A1/C13
		Hamburg	8	N110406	A1/C13
		Cologne	9	3,113,029	A1
		Cologne	10	N110416	C4.2
ACNI	2014	Kleve	11	3,646,051	A1/C4
		Kleve	11	3,646,052	C4
		Kleve	11	1,559,617	A1/C7
		Kleve	11	1,033,293	C13/A1
		Kleve	12	5,328,990	A1
		Kleve	12	5,328,991	A1
		Kleve	12	5,328,992	A1/C4
		Kleve	12	6,352,701	A1
		Kleve	13	6,352,766	A1
	2015	Kleve	12	5,328,597	A1
		Kleve	12	5,328,598	A1
		Kleve	12	6,352,777	A1
		Kleve	12	6,352,778	A1

## Discussion

In this study, we compared the influence of *Trichomonas* spp. on two *Accipiter* species breeding in Germany. While our results indicate that the Northern goshawk had a higher prevalence and a higher parasite strain diversity than Eurasian sparrowhawks, Eurasian sparrowhawks showed a higher chance of developing clinical signs of trichomonosis, independent of the identity of the infecting strain. We conclude that Northern goshawk nestlings appeared to cope better with parasite infection than Eurasian sparrowhawks, and the higher incidence of clinical signs in Eurasian sparrowhawks may pose a higher risk to sparrowhawk population viability.

### Genetic characterisation

Sequence analysis of the Fe-Hydrogenase region revealed 14 different strains in our study species from which nine were novel strains. All except one strain belonged to the types A or C. Type C showed the highest diversity in our *Accipiter* species with nine different strains, of which seven were new to the scientific community. Type A occurred with four different strains of which one was a novel strain. We found subtypes of both strain types A and C in both raptor species which solely differed from the main strains A or C in 1 to 2 point mutations, indicating that the strains have been present in German bird populations for some time, as shown by the high diversity of subtypes. The *Trichomonas* types A and C are the most common in Europe and were described for different bird species in Germany and the United Kingdom (Chi et al. 2013; Quillfeldt et al. 2018).

One exception we found in one goshawk nest from Hamburg which belonged to the ITS type III (Grabensteiner et al. 2010). III-1 was more closely related to *T. stableri* than *T. gallinae*, but we were not able to identify the species with certainty. Studies from several countries already indicated the possibility of different species of trichomonads in avian populations. Recently, Girard et al. (2014b) identified the new species *T. stableri* as the parasite involved in mortalities of band-tailed pigeons (*Patagioenas fasciata monilis*, Vigors 1839). The existence of isolates with a high similarity to the human pathogens *Trichomonas vaginalis* (Donné 1836) and *Trichomonas tenax* (Muller 1773; Dobell 1939) and the canid pathogen *Trichomonas canistomae* were reported from Europe, Brazil and the USA (Gerhold et al. 2008; Grabensteiner et al. 2010; Ecco et al. 2012; Martínez-Herrero et al. 2014, 2017; Quillfeldt et al. 2018). The occurrence of hybrid lineages was also recently reported (Alrefaei et al. 2019). The more divergent parabasalids may also cause trichomonosis-like avian diseases and *Trichomonas*-like parabasalids and the newly described species *Simplicimonas similis* (Cepicka et al. 2010) were found in birds from Brazil and the Caribbean (Ecco et al. 2012; Stimmelmayr et al. 2012). Our data suggest that several trichomonad species may circulate in German *Accipiter* populations. The presence of *T. tenax*-like and *T. vaginales*-like isolates is also described for several German bird species (Quillfeldt et al. 2018).

### Prevalence

Goshawks showed an overall higher diversity of *Trichomonas* isolates, with 13 different strains found, whereas in sparrowhawks, we identified five strains. The difference in strain diversity found between both hosts could be a result of the larger sample size in goshawks. Alternatively, or in addition, it could also indicate that specific genotypes of parasites are preferentially associated with specific host species or—because of their diet, with specific prey hosts (Martínez-Herrero et al. 2014). Goshawks mainly hunt pigeons (Kenward 2006)—the main host of *T. gallinae* (Stabler 1954)—and it is possible that pigeons harbour a greater diversity of trichomonads than other prey species, which could ultimately result in higher parasite diversity in Northern goshawks. Goshawks showed a higher prevalence of *Trichomonas* spp. and also a higher strain diversity than sparrowhawks, consistent with the idea that an increased encounter probability of the parasite can increase parasite strain diversity in the host.

Encounter probability of the parasite and of specific parasite strains may also depend on the breeding habitat of the *Accipiter* species. Whereas rural goshawks and sparrowhawks had a similar prevalence, urban goshawks had an increased prevalence. Other studies have also demonstrated a higher prevalence in urban than rural sparrowhawks (Kunca et al. 2015). Because of the loss of rural habitats for many raptor species, their traditional prey is mainly replaced by urban pigeons, so urban life is associated with an increased risk of exposure to *Trichomonas* spp. (Boal et al. 1998; Amin et al. 2014; Merling de Chapa et al. 2020). All in all, our results are consistent with the view that prevalence varies between host species because of variation in diet and breeding habitat (Martínez-Herrero et al. 2014).

### Clinical signs

While goshawks were more likely to be exposed to *Trichomonas* spp., sparrowhawks were more likely to develop clinical signs of trichomonosis. The suggestion has been made that type A isolates are normally virulent, whereas type C isolates are not as virulent (Alrefaei et al. 2019). Although the most common *Trichomonas* strains in the two *Accipiter* species belonged to both types A and C, we could not detect a difference in the proportion of individuals with clinical signs caused by specific *Trichomonas* strains. In general, there was no relationship between the identity of the infecting strain and the probability to show clinical signs of the disease. Our sampled species could have already adapted to the detected strains, and/or the strains may have become less virulent as an adaptation to facilitate their transmission (Anderson and May 1982). However, the two sampled *Accipiter* species showed a different outcome of the disease when infected with *Trichomonas* spp. which does not support the assumption that both host species are equally adapted to the parasite. Another possible explanation is that the shift to passerine birds may be the result of a genetic change in the parasite (Lawson et al. 2011a) which increased its survival time in the environment. Purple & Gerhold (2015) experimentally tested the survival time of different genotypes belonging to ITS type A and B and showed that A is more persistent in the environment than B. Therefore, the finch epidemic could be the result of an increased encounter probability of a naïve host and not the pathogenicity of the *Trichomonas* isolate itself (Lawson et al. 2011a). Even though genetic data indicate some variation between isolates, no correlation with virulence is yet established (Gerhold et al. 2008; Grabensteiner et al. 2010; Ecco et al. 2012; Stimmelmayr et al. 2012) and there is no molecular marker or molecular assay to distinguish virulent from non-virulent strains (Forrester and Foster 2008). However, Amin et al. (2012) found a correlation of peptidase activity and virulence in chicken liver cell culture and a recent study from Martínez-Herrero et al. (2019) found that membrane-associated proteins vary with virulence and some of the identified proteins are described to be potential virulence factors, similar to their orthologs in *T. vaginalis*, yet the classification of trichomonad isolates as pathogenic or non-pathogenic is still mainly based on the severity of the symptoms induced in the host (Amin et al. 2014). Our results indicate that the identification of virulent strains due to the widely used molecular markers does not seem sufficient and the susceptibility of the host seems to be an important factor to consider and studies might suffer from possible co-infections of different genotypes (Martínez-Herrero et al. 2019). While we did not find a difference in virulence of single strains, being infected with more than one strain did increase the risk to develop clinical signs of trichomonosis in our study. Sequence analysis of the ITS region has already revealed mixed infections in goshawks and sparrowhawks (Martínez-Herrero et al. 2014) as well as pigeons (Grabensteiner et al. 2010). Whereas infections with genetically diverse parasites can influence disease severity (Taylor et al. 1997; Read and Taylor 2001; Mideo 2009), we are not aware of other studies drawing a connection between infections with several parasite strains and clinical signs of trichomonosis. Further research is needed to uncover possible causes for this effect and to further identify factors linked to the virulence of the parasite.

## Conclusion

To our knowledge, this study is one of the most comprehensive investigations to date to compare parasite exposure with *Trichomonas* spp. in the same populations over several years. With our standardised methodology and appropriate population-level replication, we can draw a bigger picture of the influence one of the most important bird diseases can have on different *Accipiter* species.

While our study revealed a high diversity of *Trichomonas* strains in the two *Accipiter* species, our data support the assumption that host susceptibility has a stronger influence on the risk to express clinical signs of the disease than variation in strain virulence. High prevalence of exposure and a low proportion of visual lesions (Krone et al. 2005; Martínez-Herrero et al. 2014; Merling de Chapa et al. 2020) may indicate that goshawks had an extended evolutionary history with this parasite, leading to host-parasite co-evolution (Krone et al. 2005; Martínez-Herrero et al. 2014). Whereas rural goshawks and sparrowhawks had a similar prevalence of *Trichomonas* spp., sparrowhawks showed a higher probability to develop clinical signs. The parasite seems to be common in goshawks, whereas it is new in sparrowhawks because of the recent host expansion to passerine birds (Robinson et al. 2010; Lawson et al. 2011b), the preferred prey of sparrowhawks. Other factors are likely to influence the risk to develop clinical signs of trichomonosis, in addition to host susceptibility and parasite virulence, that might differ even between conspecific hosts such as the individuals’ genetic makeup. Further research on factors influencing the pathogenicity of the parasite strains, as well as the immune status of the infected host, is needed to assess the risk of reducing host population viability caused by trichomonosis.

## Data Availability

Data and relevant code for this research work are stored in GitHub [https://github.com/Merling-de-Chapa/tRicho]. Genetically novel identified parasite strains were submitted to GenBank (accession numbers: MW382268-MW382276).
